# Cross Talk between NOTCH Signaling and Biomechanics in Human Aortic Valve Disease Pathogenesis

**DOI:** 10.3390/jcdd1030237

**Published:** 2014-12-01

**Authors:** Richard C. Godby, Charu Munjal, Amy M. Opoka, J. Michael Smith, Katherine E. Yutzey, Daria A. Narmoneva, Robert B. Hinton

**Affiliations:** 1 Division of Cardiology, the Heart Institute, Cincinnati Children’s Hospital Medical Center, Cincinnati, OH 45229, USA; 2 Department of Biomedical Engineering, University of Cincinnati, Cincinnati, OH 45221, USA; 3 TriHealth Heart Institute, Cardio-Thoracic Surgery, Good Samaritan Hospital, Cincinnati, OH 45242, USA; 4 Molecular Cardiovascular Biology, the Heart Institute, Cincinnati Children’s Hospital Medical Center, Cincinnati, OH 45229, USA

**Keywords:** mechanobiology, NOTCH, aortic valve, biomedical engineering, mechanotransduction

## Abstract

Aortic valve disease is a burgeoning public health problem associated with significant mortality. Loss of function mutations in NOTCH1 cause bicuspid aortic valve (BAV) and calcific aortic valve disease. Because calcific nodules manifest on the fibrosa side of the cusp in low fluidic oscillatory shear stress (OSS), elucidating pathogenesis requires approaches that consider both molecular and mechanical factors. Therefore, we examined the relationship between NOTCH loss of function (LOF) and biomechanical indices in healthy and diseased human aortic valve interstitial cells (AVICs). An orbital shaker system was used to apply cyclic OSS, which mimics the cardiac cycle and hemodynamics experienced by AVICs *in vivo*. NOTCH LOF blocked OSS-induced cell alignment in human umbilical vein endothelial cells (HUVECs), whereas AVICs did not align when subjected to OSS under any conditions. In healthy AVICs, OSS resulted in decreased elastin (ELN) and α-SMA (ACTA2). NOTCH LOF was associated with similar changes, but in diseased AVICs, NOTCH LOF combined with OSS was associated with increased α-SMA expression. Interestingly, AVICs showed relatively higher expression of NOTCH2 compared to NOTCH1. Biomechanical interactions between endothelial and interstitial cells involve complex NOTCH signaling that contributes to matrix homeostasis in health and disorganization in disease.

## 1. Introduction

Aortic valve disease (AVD), broadly defined as valve dysfunction manifesting as stenosis and/or regurgitation, is a leading cause of cardiovascular morbidity and mortality. AVD affects approximately 2.5% of the general population, leading to over 20,000 deaths, 300,000 aortic valve replacement procedures, and an estimated $1 billion in direct costs each year worldwide [1–4]. As the population continues to age, these statistics are projected to worsen, making valve disease a growing public health problem [5]. At the same time, the underlying pathogenesis remains largely unknown, and the interaction between molecular and mechanical contributing factors is poorly understood.

**Figure 1:**
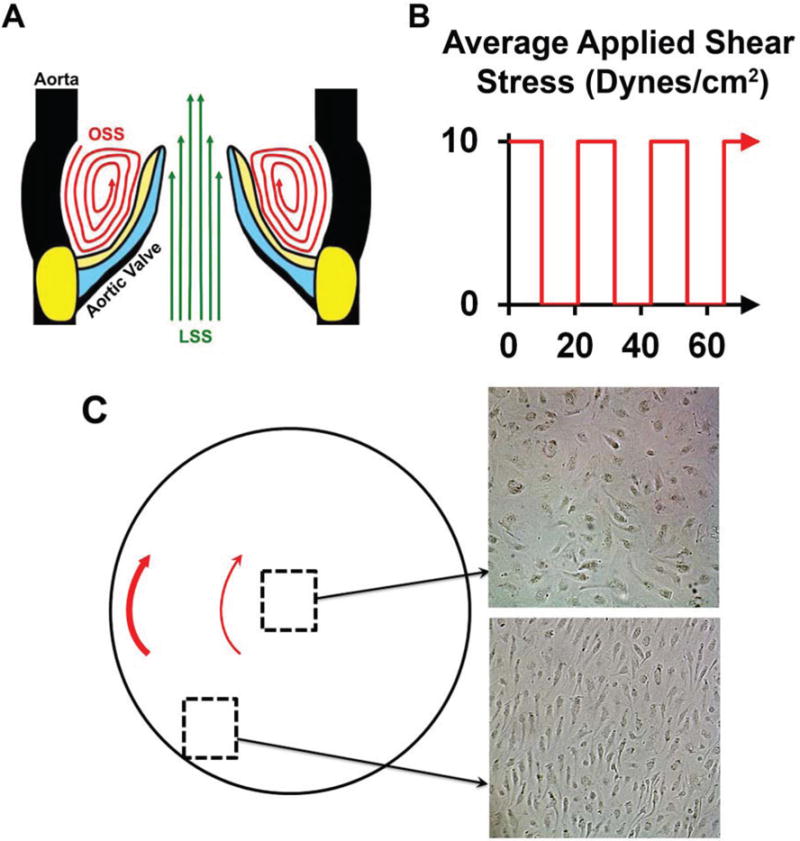
Oscillatory shear stress (OSS) experimental approach. (**A**) Regional aortic valve anatomy and hemodynamics. The aortic valve is stratified into fibrosa (yellow), spongiosa (blue), and ventricularis (black) layers. The fibrosa (arterial side of the cusp) is collagen-rich, experiences OSS (red arrows), and is the primary site of calcific lesion formation. The ventricularis (ventricular side of the cusp) is elastin-rich and experiences LSS (green arrows). (**B**) The experimental setup is designed for application of physiologic OSS at the fibrosa throughout the cardiac cycle (the x-axis showing time in seconds). Here, cells are cultured in a six-well plate attached to the orbital shaker with a controlled motion cycle. (**C**) Illustration of a single well that is in motion on the orbital shaker. The magnitude of shear stress increases with radius, as illustrated by thin (smaller stress) and thick (larger stress) red arrows. A motion regimen was chosen such that the average shear stress value was approximately 10 Dynes/cm^2^, as shown in **B**. As expected, HUVECs in the center of the well failed to demonstrate cellular alignment, indicating negligible shear stress magnitude, while HUVECs in the periphery of the well aligned parallel to the fluidic flow, consistent with larger levels of shear stress.

Anatomically, healthy aortic valve cusps are stratified into distinct and functionally interdependent layers (Figure 1A) that contain aortic valve interstitial cells (AVICs) and are covered by a monolayer of aortic valve endothelial cells (AVECs) [6]. The extracellular matrix (ECM) is stratified as follows: the fibrosa layer on the arterial aspect of the cusp is composed of mainly collagen fibers, while the ventricularis layer on the ventricular aspect of the cusp is composed primarily of elastic fibers [7, 8]. These distinct cell-matrix characteristics on either side of the valve are related to differences in the biomechanical microenvironment, where the ventricularis is predominantly exposed to laminar shear stress (LSS) only during systole, while the fibrosa is constantly loaded, experiencing oscillatory shear stress (OSS) during systole and tensile compressive forces during diastole due to backflow of blood, functioning to prevent blood pooling and ensure proper cusp coaptation [9]. Considering that calcific nodules manifest as subendothelial lesions within the fibrosa [10–12], there has been great interest in elucidating how biomechanics impacts ECM composition, valve homeostasis, and pathobiology.

Congenital aortic valve malformations such as bicuspid aortic valve (BAV) underlie the majority of cases of calcific AVD, and can be caused by loss of function mutations in NOTCH1 [13,14]. Previous studies demonstrated an important role of NOTCH signaling in valvulogenesis. NOTCH signaling involves four receptors (NOTCH1–4), which regulate transcriptional programs largely via transcriptional repressors such as HES1 and HEY2 [15–18]. Previous *in vitro* and *in vivo* studies have established that inhibition of Notch signaling in mammalian AVICs resulted in calcification [18–20]. NOTCH signaling also plays a role in arterial remodeling and has direct effects upon elastin [21–24]. Interestingly, NOTCH1 signaling has been described as primarily localized to the endothelium of the ventricularis, giving it a spatial association with elastic fibers, while NOTCH2 activity has been described throughout AVICs [25, 26]. However, the relationship between NOTCH signaling and elastin in the context of the aortic valve is still unknown.

In genetically predisposed and/or structurally defective aortic valve tissue, pathological processes can also be triggered by local perturbations in physiologic OSS. For example, the fibrosa of BAVs is exposed to more turbulent OSS, where the flow patterns are reminiscent of those around arterial bifurcations, which have been linked to atherosclerosis [27–31]. Recently, several studies have described important ECM and biomechanical differences between the arterial and ventricular sides of the cusp [32–34], suggesting a possible connection between the OSS-exposed arterial side of the valve and disease manifestation [35,36]. The role of OSS as it pertains to NOTCH dysregulation is unknown.

AVICs are a heterogeneous cell population that is influenced by the inherently dynamic homeostasis of the ECM biomechanical environment due to constant tissue movement that can be progressively maladaptive in disease [34,37]. A monolayer of AVECs directly responds to OSS *in vivo*, and AVICs are responsive to both mechanical stimuli and AVEC signaling in both health and disease [34,37–39]. How AVECs inform AVICs through mechanotransductive processes is of increasing interest [40], and the established role of NOTCH signaling in AVECs and AVICs in health and disease suggests NOTCH plays a crucial role in this process [29,41,42]. The important role of AVEC-AVIC communications in valve development and disease is further supported by reports that VEGFA, which is a major factor in vascular remodeling, can act in opposition to NOTCH in the context of valve endothelial to mesenchymal transformation [43–45].

Blood flow and hemodynamic measures play an important role in healthy valvulogenesis [46,47]. For instance, human aortic valves show progressively robust elastogenic processes within the ventricularis layer of a valve, which is consistent with a role for mechanobiological signals in valvulogenesis [39,48,49]. AVD is a progressive process that is mediated by aberrant reactivation of developmental pathways associated with valve development [10–12], and overt calcification in the advanced disease state is an active, rather than passive, process [50], suggesting it can be modified. However, the precise interactions between molecular programs and mechanical factors involved in regulating valve development and homeostasis remain largely unknown.

The objective of this study was to develop a feasible and reliable experimental approach to exploring the role of NOTCH in the mechanobiology of human AVICs. We hypothesized that NOTCH loss of function would alter AVIC responses to OSS in a manner consistent with progressive disease processes. Here, for the first time, we describe the effects of NOTCH loss of function and OSS in cultured AVICs isolated from healthy and diseased tissue. These findings may have implications for valve tissue engineering efforts, as well as the identification of new molecular targets to treat AVD.

## 2. Materials and Methods

### 2.1. Cell Culture and Treatment

Healthy AVICs were harvested from human hearts (*n* = 3) received through the National Disease Research Interchange program. These tissues were obtained from donors (two female and one male) under the age of 40 years old (26–38 years) with normal cardiac structure and function, including functional tricommissural aortic valve morphology, who died of non-cardiac causes and experienced a warm ischemia time of less than six hours. Diseased AVICs were harvested from human valves (*n* = 3) procured during valve replacement surgeries from patients (one female and two male) under the age of 40 years old (14–34 years); all diseased valves had BAV and fibrotic aortic stenosis. Following tissue digestion with collagenase, AVICs were cultured in M199 media (Life Technologies) supplemented with 10% FBS and penicillin/streptomycin, as previously described [24]. Cell phenotype was confirmed by negative vWF and MF-20 staining after passage 4 to rule out contamination by AVECs or myocardial cells (data not shown). Human umbilical vein endothelial cells (HUVECs) were procured from American Type Culture Collection and cultured in F12 media supplemented with 10% FBS, Heparin, ECGF, and penicillin/streptomycin (Sigma, Santa Clara, CA, USA). Cells between passages 5–10 were used in all experiments. These studies were approved by the Institutional Review Board at Cincinnati Children’s Hospital Medical Center.

Cells were cultured on six-well plates, followed by one of the following treatments: NOTCH inhibition using *DAPT treatment*: AVICs and HUVECs were treated for seven days with the γ-secretase inhibitor DAPT (N-[N-(3,5-difluorophenacetyl-L-alanyl)]-S-phenylglycine t-butyl ester, Calbiochem) reconstituted in DMSO, and used at a final concentration of 50 μM in complete media, or with a vehicle control using an equivalent volume of DMSO, as previously described [18,19,51]; *Osteogenic treatment*: AVICs were treated for 10 days with complete media containing 10 mM β-glycerophosphate, 60 μM ascorbic acid-2-phosphate, and 1 μM dexamethasone [52]; or *Elastin-derived peptide (EDP) treatment*: AVICs were treated for 72 h with soluble EDP (10 μg/mL, Elastin Products Company) in complete media or vehicle treated, as previously described [53].

### 2.2. Oscillatory Shear Stress

AVICs seeded in six-well plates containing either complete media supplemented with DAPT (2 mL media/well) or the vehicle control were placed on a Fisher Scientific Clinical Rotator (14-251-200), with a 0.95 cm radius of orbit, which was set to 200 revolutions per minute. OSS was applied for 10 s then ceased for 12 s in order to mimic a systolic:diastolic ratio of 0.8 to approximate the cardiac cycle (Figure 1B) [54], using an electrical circuit interrupter (CT-1 Adjustable Cycle Timer; Innovative Grower) (Supplement; Videos S1–S3). Cells were exposed to OSS for 24 h. Assuming that ramping is negligible and given certain properties of the media (η_media_ (0.00101 kg/(m × s)), ρ_media_ (997.3 kg/m^3^), η_air_ (1.7894 × 10^−5^ (1/(m × s))) and ρ_air_ (1.225 kg/m^3^) [55]), this setup results in an estimated average radial shear stress of 10 Dynes/cm^2^, approximating physiologic OSS [55–57]. To validate this setup, HUVECs were subjected to the OSS as described above. HUVECs in the center of the well failed to demonstrate cellular alignment, indicating negligible shear stress magnitude at this location, while HUVECs in the periphery of the well aligned parallel to fluidic flow, consistent with larger levels of shear stress (Figure 1C). Cellular alignment was defined as a majority of cells oriented in the same plane within a total of 30 degrees variation (*vs.* random) [42,57–60].

### 2.3. Alizarin Red Staining

AVICs were processed and stained with 2% Alizarin Red as previously described [51]. Images were captured using a Nikon Diaphot 300 microscope and NIS Elements software.

### 2.4. RNA Isolation and Real-Time Quantitative RT-PCR

Total RNA was isolated from cellular monolayers using 500 μL/well of TRIzol reagent (Invitrogen, Waltham, MA, USA). cDNA was generated (Applied Biosystems) from 1 μg of total RNA. Forward and reverse primers for real-time quantitative RT-PCR (qRT-PCR) were created (Supplement: Table S1). A total of 10 ng of synthesized cDNA was used per experiment (Bio-Rad), as previously described [61]. Gene expression levels were normalized to 18S rRNA and reported as mean ± standard deviation.

### 2.5. VEGF-A ELISA

Following treatment with osteogenic media or EDP, the supernatant was collected and kept at −80°C for VEGF-A ELISA, which was performed according to manufacturer’s instructions (R&D Systems).

### 2.6. Statistics

Statistical analyses were performed using analysis of variance (ANOVA) and a post-hoc test with Bonferroni corrections (SPSS). Significance was considered at *p* < 0.05.

## 3. Results

### 3.1. NOTCH Loss of Function in Human AVICs Leads to Calcifications in Vitro

**Figure 2:**
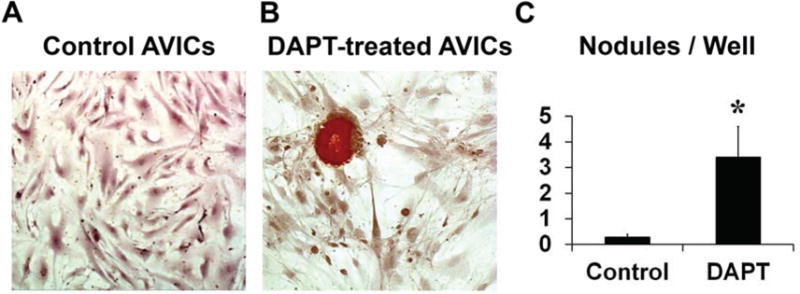
NOTCH loss of function in healthy human AVICs leads to calcific nodules *in vitro*. (**A**) Healthy AVICs treated with the vehicle control did not demonstrate calcification when stained with Alizarin Red. (**B**) In contrast, DAPT-induced NOTCH loss of function in healthy AVICs demonstrated formation of calcific nodules. (**C**) Statistical analysis demonstrated a significantly larger number of calcifications per well for the DAPT group, as compared to the vehicle control group. Magnification: 20×.

To confirm that AVIC calcification can be induced in human AVICs by NOTCH loss of function, healthy AVICs were treated with DAPT. The results showed formation of calcific nodules in the DAPT group, in contrast to no evidence of calcification in AVICs treated with the vehicle control (Figure 2). Statistical analysis demonstrated a significantly larger number of calcification nodules in AVICs treated with DAPT when compared to AVICs treated with the vehicle control (Figure 2C), consistent with previous *in vitro* studies using non-human AVICs and diseased human AVICs [18,19,51].

### 3.2. NOTCH Loss of Function Leads to Decreased ELN and α-SMA Expression in Human AVICs

**Figure 3:**
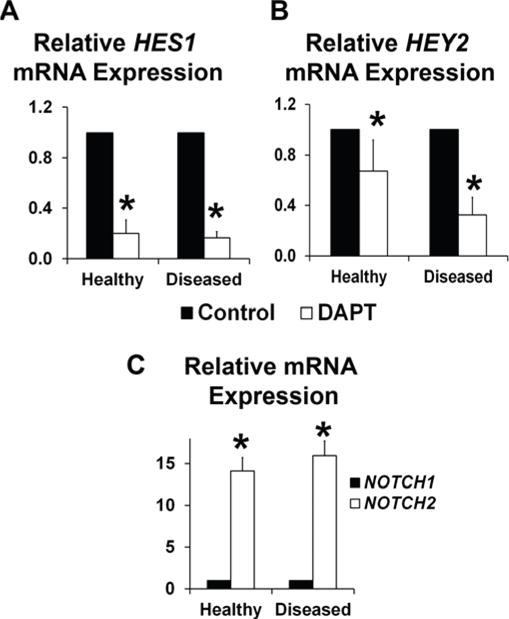
DAPT treatment results in similar NOTCH loss of function responses by healthy and diseased human AVICs. (**A**,**B**) DAPT treatment resulted in significant decrease in HES1 and HEY2 expression, as measured by qRT-PCR, for both healthy and diseased AVICs. (**C**) No difference in gene expression for NOTCH1 and NOTCH2 receptors was observed between healthy and diseased AVICs, while levels for NOTCH2 expression was significantly greater than those for NOTCH1 expression in both cell populations. Gene expression was normalized for each group.

To elucidate the mechanism of how disease affects cell-ECM biomechanical interactions, both healthy and diseased AVICs were subjected to NOTCH LOF. Baseline expression of HES1 and HEY2 was not significantly different between healthy and diseased AVICs (data not shown). No difference in relative gene expression for HES1 and HEY2 was observed between healthy and diseased AVICs (1.20 ± 1.34, 1.92 ± 1.64, respectively; *p* = 0.40, *p* = 0.19, respectively), where expression was normalized to 1 in healthy AVICs. DAPT treatment resulted in a significant reduction of the downstream NOTCH effector genes HES1 and HEY2 in both cell populations (Figure 3), consistent with previous studies [16,18,19,62]. Our results showed a more robust DAPT-induced inhibition of HES1, as compared to HEY2; therefore, in subsequent experiments, HES1 was used as a surrogate for NOTCH signaling. Levels for NOTCH2 expression were significantly greater than those for NOTCH1 expression in both healthy and diseased cell populations (Figure 3C). No difference in relative gene expression for NOTCH1 and NOTCH2 receptors was observed between healthy and diseased AVICs (0.84 ± 0.36, 0.92 ± 0.30, respectively; *p* = 0.24, *p* = 0.35, respectively), where expression was normalized to 1 in healthy AVICs.

**Figure 4:**
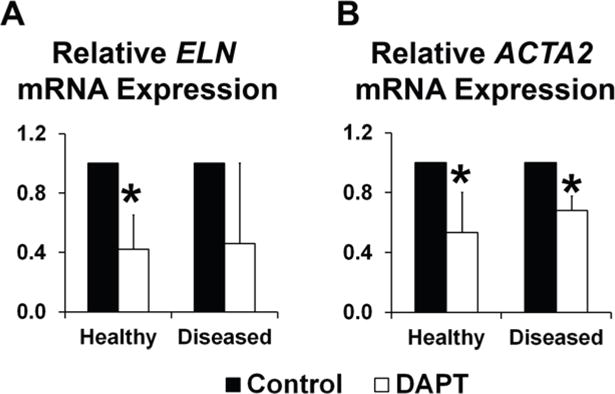
NOTCH loss of function leads to decreased ELN and ACTA2 expression in human AVICs. (**A**) DAPT treatment resulted in a significant decrease in ELN expression in healthy AVICs, while the trend in diseased cells was not significant. (**B**) DAPT-induced NOTCH loss of function resulted in significant reduction in levels of ACTA2 gene expression in both healthy and diseased AVICs. Gene expression was normalized for each group.

Because NOTCH signaling has been demonstrated in the ventricularis layer [49,63], we explored the effects of NOTCH LOF on elastin (ELN) and α-SMA (ACTA2) expression, two associated markers specific to the ventricularis. In healthy AVICs, ELN expression was significantly decreased in response to DAPT-induced NOTCH LOF (Figure 4A). Diseased AVICs also showed decreased ELN, but this failed to reach statistical significance, possibly due to tissue heterogeneity (*p* = 0.08). In both healthy and diseased AVICs, ACTA2 expression was decreased significantly with NOTCH LOF (Figure 4B), suggesting that NOTCH signaling contributes to the regulation of elastic fiber homeostasis in the ventricularis layer.

### 3.3. OSS and NOTCH Provide a Complex Regulation of ELN and ACTA2 Expression in Human AVICs

**Figure 5:**
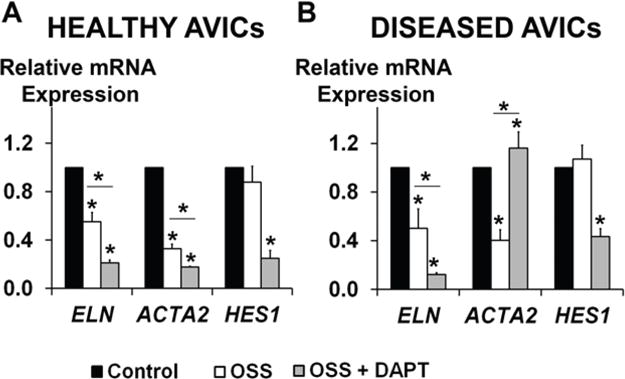
ELN and ACTA2 expression changes in response to OSS and NOTCH LOF in human AVICs. In both healthy (**A**) and diseased (**B**) AVICs, OSS exposure resulted in significantly decreased ELN and ACTA2 expression, while no effect of OSS on HES1 was observed. In healthy AVICs, a combination of OSS and NOTCH LOF (DAPT treatment) resulted in further inhibition of ELN and ACTA2 expression, and a decreased HES1 expression. In contrast, in diseased cells, a combination of OSS and NOTCH LOF had the opposite effect on ACTA2 than either OSS or NOTCH LOF treatment alone, resulting in significantly higher ACTA2 expression levels for OSS with NOTCH LOF group, as compared to the controls. At the same time, the combination of OSS and NOTCH LOF in the diseased cells resulted in further decreases in ELN and HES1 expression levels as compared to NOTCH LOF alone, which was similar to the responses in healthy cells.

When subjected to OSS, neither healthy nor diseased AVICs demonstrated any change in cellular alignment (data not shown), indicating a different phenotype from established stress-sensitive HUVECs. In both healthy and diseased AVICs, OSS exposure resulted in significantly decreased ELN and ACTA2 expression, while no effect of OSS on HES1 was observed (Figure 5). In healthy AVICs, a combination of OSS and NOTCH LOF resulted in further inhibition of ELN and ACTA2 expression, and decreased HES1 expression. In contrast, in diseased cells, a combination of OSS and NOTCH LOF had the opposite effect on ACTA2 than either OSS or NOTCH LOF alone, resulting in significantly higher ACTA2 expression levels for the OSS with NOTCH LOF group, as compared to the controls. Moreover, the combination of OSS and NOTCH LOF in the diseased cells resulted in decreased ELN and HES1 in comparison to NOTCH LOF alone, which was similar to the responses in healthy cells. These results suggest that NOTCH LOF may play a role in disease by activating a subset of myofibroblast-like AVICs (ACTA2-positive) in response to OSS that may initiate loss of ECM homeostasis and early disease processes. Therefore, OSS may play a secondary role in the context of abnormal NOTCH signaling, effectively advancing the disease process.

### 3.4. Disease Differentially Affects VEGFA Gene and Protein Expression Levels

**Figure 6:**
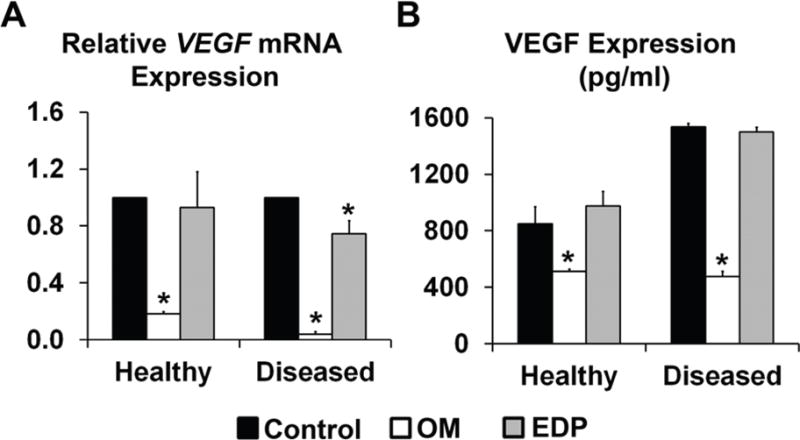
Disease differentially affects VEGFA gene and protein expression levels. In healthy AVICs, treatment with osteogenic media resulted in significant inhibition of both VEGF mRNA (**A**) and protein (**B**) expression, while no effect was observed for EDP. Interestingly, while mRNA levels for VEGFA were similar between healthy and diseased AVICs, the basal levels of VEGFA protein in the diseased group were significantly higher as compared to healthy controls. Further, treatment of diseased cells with osteogenic media resulted in stronger inhibition of VEGFA mRNA and protein expression, as compared to the healthy group. In the disease AVICs, EDP treatment led to a small decrease in VEGFA mRNA, but not protein expression levels, as compared to controls.

Because NOTCH1 mutations have been shown to cause AVD, elastic fiber fragmentation has been implicated in the development of AVD [64–68]. Because VEGFA has been established as a major factor in valve development and disease, and has been shown to act in opposition to NOTCH in the context of valve endothelial to mesenchymal transformation [43–45], we examined the effects of NOTCH LOF on VEGF gene and protein expression in cells treated with osteogenic media or EDP. Since experiments from the current study show that NOTCH LOF resulted in decreased ELN, and EDP is known to induce calcification through smooth muscle cell activation [64], we examined the effects of EDP. In healthy AVICs, treatment with osteogenic media resulted in significant inhibition of both VEGF mRNA and protein expression, while no effect on EDP was observed (Figure 6). Interestingly, while mRNA levels for VEGFA were similar between healthy and diseased AVICs, the basal levels of VEGFA protein in the diseased group were significantly higher as compared to healthy controls [69], suggesting important pathologic differences between transcriptional and translational regulatory mechanisms of VEGFA [70,71], such as post-translational modification, which impacts receptor function [72] and therefore warrants further investigation. Further, treatment of diseased cells with osteogenic media resulted in stronger inhibition of VEGFA mRNA and protein expression, as compared to the healthy group. In diseased AVICs, these preliminary findings suggest that EDP treatment leads to a modest decrease in VEGFA mRNA, but not protein levels compared to controls.

### 3.5. NOTCH Loss of Function Leads to Cellular Misalignment in HUVECs in Response to OSS

**Figure 7:**
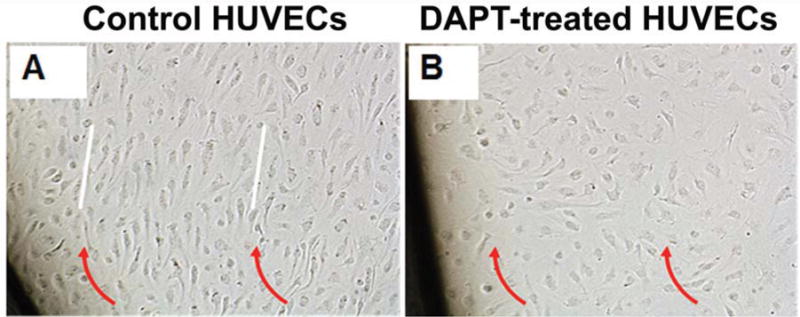
NOTCH loss of function abolishes cellular alignment in HUVECs in response to OSS. (**A**) Exposure of HUVECs to OSS resulted in a marked alignment of HUVECs (white lines) parallel to the direction of fluidic flow (red arrows) when treated with vehicle control; (**B**) HUVEC response to OSS was abolished in the presence of DAPT. Magnification 10×.

Because of the major role of endothelial cells in mechanotransduction in response to OSS [73], and the known deleterious effect of NOTCH LOF on AVIC function in AVD [19], we sought to determine the role of NOTCH LOF in endothelial cells in response to OSS. Because reliable methods for isolating AVECs were not available at the time of the study, HUVECs were used as an alternative source of endothelial cells. While OSS alone induces a marked HUVEC alignment, this response was abolished in the context of NOTCH LOF, but not the vehicle control (Figure 7). In contrast, exposure to OSS or OSS with NOTCH LOF did not induce any detectable preferential cell alignment in AVICs (data not shown), suggesting NOTCH signaling is necessary for cellular alignment in endothelial but not interstitial cells, consistent with the role of endothelium as the first frontier in biomechanical signal transduction across the valve surface layer to the AVICs. Together, these findings suggest that OSS regulates valve ECM homeostasis in the context of local hemodynamics in part via a NOTCH-mediated mechanotransductive mechanism between AVICs and the endothelium.

## 4. Discussion

The findings of this study present evidence for the first time of the role of NOTCH in the mechanobiology of healthy and diseased human AVICs. These results support the concept that important endothelial to interstitial cell communications are mediated in part by mechanotransductive processes impacted by hemodynamics and local stresses. These findings suggest that NOTCH LOF results in disruption of elastic fiber ECM homeostasis and activation of ACTA2, and OSS predisposes valve tissue to maladaptive responses. These findings potentially inform the emerging interest in mechanisms of valve homeostasis and disease progression.

Loss of function mutations in NOTCH1 cause calcific AVD, but loss of function can be realized in a number of different ways. DAPT globally inhibits NOTCH signaling, including NOTCH1 and NOTCH2 receptors, and results in calcification, suggesting that multiple NOTCH pathways contribute to disease pathogenesis and the role of NOTCH2 may be underestimated. It has been shown that patients with Alagille syndrome, caused by mutations in JAG1 or NOTCH2, develop calcific AVD [74]. Notch signaling facilitates epithelial-to-mesenchymal transition during valvulogenesis and contributes to ECM stratification and postnatal maturation and homeostasis [25,75–77]. AVICs demonstrate higher levels of NOTCH2 than NOTCH1. While NOTCH1 and NOTCH2 converge upon the same downstream effector genes, and therefore are to some degree functionally redundant, they are differentially regulated as evidenced in part by different expression patterns in the developing and mature cardiac outflow tract [78,79]. Our findings suggest that biomechanical forces may influence these pathways, impacting both homeostasis and disease.

One intriguing difference between healthy and diseased AVICs in this study was that NOTCH LOF and OSS together potentiate the activation of ACTA2 in diseased AVICs, while either of those factors alone resulted in downregulation of ACTA2. ACTA2 is the gene that encodes α-SMA; therefore, these results suggest that a combination of NOTCH LOF and OSS in diseased AVICs may lead to activation of a subset of AVICs consistent with a myofibroblast-like response and/or a compensatory response to NOTCH LOF [80]. Because NOTCH signaling is involved in cardiac neural crest cell differentiation into smooth muscle cells, it is tempting to speculate that regulation of NOTCH1 and NOTCH2 play a significant role in “activation” of AVICs in pathologic responses.

AVICs are notoriously difficult to work with *in vitro*. Because these studies were performed with human AVICs, tissue heterogeneity may have hindered certain experiments from yielding statistically significant differences because diseased valves have significant areas of ostensibly normal tissue potentially diluting the presence of abnormalities. While NOTCH1 mutations account for a very small subset of BAV cases (<2%), and BAV is generally considered multifactorial, it is important to note that the patients included in this study were not genotyped [13,81]. Presently, there is inadequate information to warrant clinical genetic testing, but we hope that ultimately genotypic variability will predict phenotypic variability in a clinically beneficial way. Another limitation of this study is the use DAPT as a non-specific inhibitor of NOTCH signaling, as this may lead to significant off-target effects such as the inhibition of the Erb-B4 signaling pathways [82]. Additionally, valvular endothelial cell isolation is technically difficult and not logistically feasible for large-scale studies; however, recent literature has begun to develop new and promising protocols aimed at achieving pure isolations [83]. Moreover, HUVECs have been shown to demonstrate a biological interplay with valvular interstitial cells *in vitro*, serving as a widely accepted surrogate [73]. Although HUVECs provide much insight into endothelial biology, they are nonetheless inherently distinct from AVECs [29,42,84–86]. These cell culture limitations should be considered when studying human valve mechanobiology *in vitro*.

**Figure 8:**
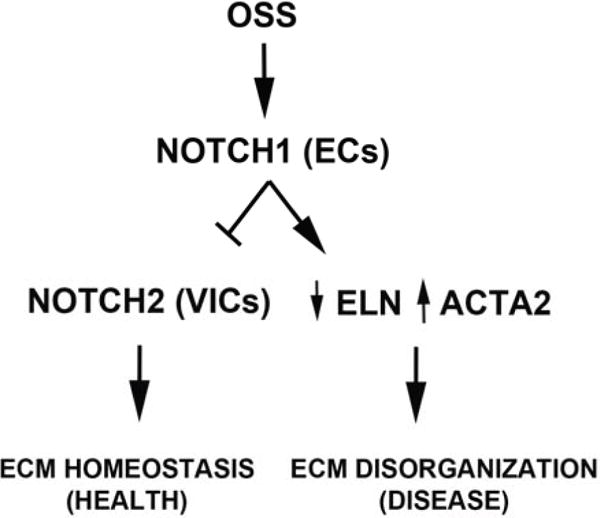
Proposed model of NOTCH signaling dysregulation and OSS. In health, oscillatory shear stress (OSS) acts directly on NOTCH1 signaling in AVECs to promote ECM homeostasis in the interstitium in cooperation with NOTCH2. In disease, NOTCH LOF results in decreased ELN and increased ACTA2 and loss of ECM organization.

Our results demonstrated that HUVECs require NOTCH signaling for cellular alignment when subjected to physiologic OSS, while this mechanism does not appear to be necessary for the AVICs’ response. These results underscore the important role of the biomechanical microenvironment in regulating signaling in stress-sensing cells and are consistent with previous reports that AVECs differ from vascular endothelial cells in their response to shear stress, as well as that AVECs from the ventricular side of the valve differ in their responses to shear stress from those of the fibrosa side [29,42,84–86]. The relationship between AVECs and AVICs has been studied in the context of NOTCH signaling and calcific AVD [73], but the role of biomechanical forces is largely unknown. The findings of the current study suggest that laminar sheer stress (LSS) and oscillatory shear stress (OSS) may have different effects in tissue predisposed to NOTCH LOF, implying that the manifestation of overt calcific AVD requires both processes (Figure 8). Our results showed OSS-induced downregulation of markers of the ventricularis side of the aortic valve in human AVICs, suggesting that there are protective elements within the ventricularis. Overall, our findings indicate that mechanotransductive processes between endothelial and interstitial cells may involve complex NOTCH signaling and contribute to ECM homeostasis.

In this study, we have presented a novel experimental system for studying valve cell responses to biomechanical stimulation. Biomechanical microenvironments are crucial for valve tissue homeostasis and tissue integrity over time [10,87–91]. The hemodynamics underlying AVD pathobiology have been extensively studied and the role of abnormal stresses established [14,92]. Previous *in vitro* and *ex vivo* studies have used parallel plate flow chambers and cone-and-plate rheometers to model hemodynamics at the fibrosa of the aortic valve [28,29,42,84,85,93]. The physiologic OSS experienced by the fibrosa side of the aortic valve has been measured to peak at approximately 10 dynes/cm^2^, which can be achieved with an orbital shaker, and computational modeling has confirmed that the average wall shear stress is approximately 10 Dynes/cm^2^ [55,84,85,93]. The approach used for this study was made more physiologically relevant by introducing an electrical circuit interrupter to periodically turn the orbital shaker on and off to recapitulate the pulsatile nature of the cardiac cycle. One advantage of the orbital shaker approach is that it allows for simultaneous, rather than sequential, treatment with multiple biochemical modulators. More studies that combine biomechanical and molecular approaches to valve structure and function are needed to understand tissue homeostasis and disease progression.

## 5. Conclusions

NOTCH LOF in the setting of OSS activated ACTA2 expression in AVICs and significantly decreased ELN content, consistent with the hypothesis that NOTCH dysregulation combined with OSS plays a role in AVD processes. The contribution of laminar (LSS) and oscillatory (OSS) shear stress to pathology may be related to spatial considerations in the context of a malformed valve and/or molecular considerations in the context of impaired AVEC–AVIC mechanotransduction on one side of the valve. AVICs have a preponderance of NOTCH2 receptor expression, while AVECs are known to be rich with NOTCH1, emphasizing the complexity of NOTCH signaling in valve tissue and suggesting there may be a coordinated process involving both receptors in disease manifestation (Figure 8). The approach to physical testing reported in this study may enhance future efforts to delineate molecular mechanisms underlying both LSS and OSS. A better understanding of valve homeostasis is crucial for identifying the mechanisms of AVD progression and setting new therapeutic targets.

## Supplementary Material

Supplementary Materials

Video S1

Video S2

Video S3
