# Supplementary material for: Cross Talk between NOTCH Signaling and Biomechanics in Human Aortic Valve Disease Pathogenesis
Source: J Cardiovasc Dev Dis. Author manuscript; Available in PMC 2018 Mar 16. (PMC5856658; doi:10.3390/jcdd1030237)
Supplement: Supplementary Materials [file NIHMS948028-supplement-Supplementary_Materials.pdf]

Supplementary Materials

Video S1. Video demonstrating the electrical circuit interrupter used to turn the orbital shaker on and off in order to recapitulate the pulsatile nature of the cardiac cycle.

Video S2. Video demonstrating the fluid dynamics experienced from the motion of an orbital shaker.

Video S3. Video demonstrating how the orbital shaker was setup inside of a cell culture incubator.

Table S1. Forward and reverse primers for real-time quantitative RT-PCR.

Target	Forward	Reverse
18S rRNA	5'-CTCTTAGCTGAGTGTCCCGC-3'	5'-CTGATCGTCTTCGAACCTCC-3'
ACTA2	5'-CCTGACCCTGAAGTACCCGA-3'	5'-GTCCAGCACGATGCCAGTT-3'
ELN	5'-ACTTGGAGGAGTGGCAGCAAGAC-3'	5'-AGGGGCATGGGATGGGGTTACA-3'
HES1	5'-CTGCTACCCCAGCCAGTGTCAA-3'	5'-TGGAATGCCGCGAGCTATCTTTCT-3'
HEY2	5'-GGCAACAGGGGGTAAAGGCTACT-3'	5'-TGGCGCAAGTGCTGAGATGAGA-3'
NOTCH1	5'-TCTGCCTGACACCCCTGGACAA-3'	5'-CCTGCTGGCACGATTTCCCTGA-3'
NOTCH2	5'-CCCCCACAATGGACAGGACAGTT-3'	5'-TGGAGTACAGGAGGCGAAGGCA-3'
VEGFA	5'-TACCTCCACCATGCCAAGTGGTC-3'	5'-ATGTCCACCAGGGTCTCGATTGG-3'
